# Dissection spontanée et isolée du tronc cœliaque

**DOI:** 10.11604/pamj.2015.22.213.7803

**Published:** 2015-11-09

**Authors:** Melek Ben Mrad, Nizar Elleuch

**Affiliations:** 1 Service de Chirurgie Cardio-vasculaire, Hopital La Rabta, Faculté de Médecine de Tunis, Université Tunis El Manar, Tunisie

**Keywords:** Dissection, tronc coeliaque, angioscanner, Dissection, celiac trunk, CT angiography

## Image en medicine

Les dissections spontanées des artères viscérales sont des entités rares. Nous rapportons le cas d'une dissection spontanée isolée du tronc cœliaque chez un patient âgé de 47 ans, tabagique, hypertendu depuis 10 ans sous bithérapie, qui a consulté les urgences pour épigastralgies intenses évoluant depuis deux jours, résistantes aux antalgiques habituels et aux inhibiteurs de la pompe à protons. L’échographie abdominale est sans particularité. La fibro-oeso-gastro-duodénale est revenue normale. Le bilan pancréatique est normal. L'examen clinique était normal à part un pic hypertensif à 185/115 mmHg. Le diagnostic a été réalisé grâce à l'exploration tomodensitométrique qui objectivait une dissection isolée du tronc cœliaque, le reste de l'arbre artériel était normal. Le patient était hospitalisé, mis sous inhibiteur calcique à la pousse seringue électrique et sous héparine à bas poids moléculaire. L’évolution était bonne avec disparition des douleurs. Le patient était mis sortant au bout de 5 jours sous anti-agrégants plaquettaires. Un contrôle scanographique annuel est prévu pour déceler une possible transformation anévrysmale.

**Figure 1:**
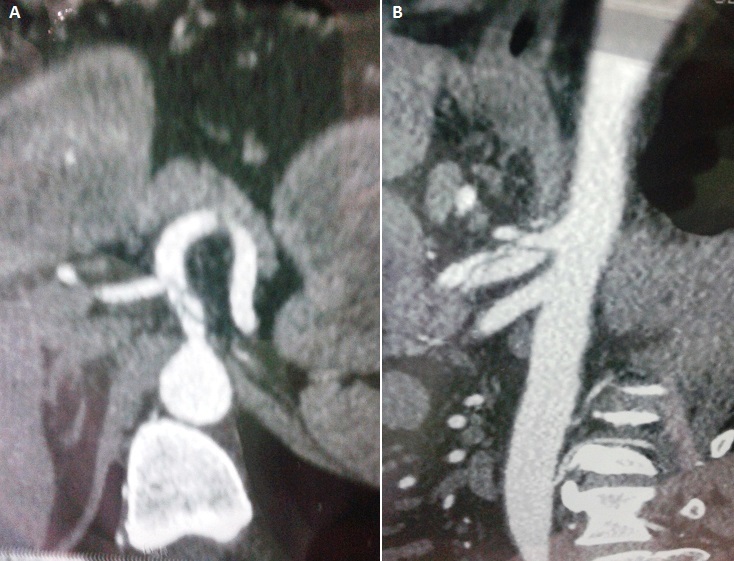
Images d'angioscanner de l'aorte abdominale: A) coupe transversale montant la dissection du tronc cœliaque; B) reconstruction latérale montrant la dissection du TC et l'intégrité de l'artère mésentérique supérieure

